# Evaluation of a bronchoscopy guidance system for bronchoscopy training, a randomized controlled trial

**DOI:** 10.1186/s12909-019-1824-3

**Published:** 2019-11-21

**Authors:** Andreas Follmann, Carina Barbosa Pereira, Julia Knauel, Rolf Rossaint, Michael Czaplik

**Affiliations:** Department of Anesthesiology, University Hospital RWTH Aachen, Pauwelsstr. 30, D-52074 Aachen, Germany

**Keywords:** Bronchoscopy, Guidance system, Bronchoscopy training, Electromagnetic navigation bronchoscopy

## Abstract

**Background:**

Conventional training in bronchoscopy is performed either on patients (apprenticeship model) or phantoms. While the former is associated with increased rate of patient complications, procedure time, and amount of sedation, the latter does not offer any form of feedback to the trainee. This paper presents a study which investigates whether a bronchoscopy guidance system may be a helpful tool for training of novice bronchoscopists.

**Methods:**

A randomized controlled study with 48 medical students was carried out with two different groups (control and test group, each *N* = 24). Whereas the control group performed a conventional bronchoscopy on phantom the test group carried out an Electromagnetic Navigation Bronchoscopy (ENB) for tracking of the bronchoscopal tip in the bronchial system. All volunteers had a common task: to perform a complete and systematic diagnostic bronchoscopy within 10 min.

**Results:**

The test group examined significantly more lobes than the control group (*p* = 0.009). Due to the real-time feedback of the system, all students of test group felt more confident having analyzed the entire lung. Additionally, they were unanimous that the system would be helpful during the next bronchoscopy.

**Conclusions:**

In sum, this technology may play a major role in unsupervised learning by improving accuracy, dexterity but above all by increasing the confidence of novices, students as well as physicians. Due to good acceptance, there may be a great potential of this tool in clinical routine.

## Background

Since its first introduction in the 1960s, flexible bronchoscopy has become a well-established and relatively safe method for a wide range of diagnostic and therapeutic interventions in the bronchial system. This technique is commonly used in the diagnosis or detection of lung cancers, chemical and thermal burns of the airway as well as hemoptysis, diaphragmatic paralysis, interstitial lung diseases, chest trauma, among others. Therapeutically, flexible bronchoscopy can be used, e.g., for bronchial washing, foreign body removal, brachytherapy, endobronchial lung volume reduction and aspiration of cysts (either bronchial, mediastinal or pericardial) [1–3].

Despite all medical benefits, the examination method itself can pose a threat to the patient’s health. Typical complications are hypoxemia, cardiac arrhythmias, myocardial ischemia, bleeding, pneumothorax, fever, infection and injury or perforation of the tracheobronchial system [3]. Hypoxemia, for instance, is considered to be correlated with the duration of the procedure [4]. Apart from the complications directly caused by the examination, patients can also be harmed by the side effects of local anesthesia or sedation [5, 6].

In 2013, Stather et al. published a paper studying on different aspects of the influence of trainees/non-trainees during diagnostic bronchoscopy. The results demonstrated distinct differences between the two groups (trainees and non-trainees) regarding procedure length (58.32 min vs 37.69 min, *p* = 0.001), administration of Propofol (178.3 mg vs 137.1 mg, *p* = 0.002) and complications rate (4.7% vs 1.1%, *p* = 0.076) [5]. Quelette et al. showed that patients undergoing bronchoscopy performed by novice bronchoscopists, more specifically first-year fellows during the first academic trimester, had a significantly increased complication rate [7]. Furthermore, inexperienced/novice bronchoscopists can a priori be expected to have a higher risk of underdiagnosing. Although this cannot be classified as a complication, consequences for the patient can be severe, e.g. due to a postponed diagnosis of lung cancer [5, 8].

Patient safety is a crucial requirement for the healthcare system which makes a proper training of the clinical staff mandatory. However, training on patients is considered increasingly unethical [9]. [10–12] In daily practice, there is also little time for proper training of novice bronchoscopists by experienced examiners, thus supervised training is difficult to implement. As a result, the apprenticeship model is being replaced by training on phantoms and simulators.

Using phantoms for training in bronchoscopy is recommended in several guidelines [13–15]. Flexible bronchoscopy is the most wanted procedure for simulation-based training [16]. Studies have shown that simulations not only to improve performance [17–19], but also to increase patient comfort [2, 20] and lead to shorter procedure time [21]. [22] In addition, a reduction of error rates is reported [23]. However, a major drawback is the absence of feedback information: An inexperienced examiner can be expected to lose orientation during the examination without supervision or proper training. One possibility for giving feedback could be the use of spatial information by means of Electromagnetic Navigation Bronchoscopy (ENB) [24]. This new method has already been evaluated in several studies, e.g. in combination with CT-Scans for locating pathological lesions in the bronchial system [25, 26]. Using an electromagnetic field, ENB keeps track of the bronchoscopal tip in the bronchial system. Applied to a bronchoscopic phantom, (novice) bronchoscopists receive a real-time feedback through a tracking system visualized on a monitor showing the tip’s current position in the lung.

This study aims to investigate whether a bronchoscopy guidance system may be a helpful tool for training of novice bronchoscopists. For this purpose, a randomized study was carried out at RWTH Aachen University in order to compare and analyze the performance of a control group (performing a conventional bronchoscopy) and a test group (performing tracking-based bronchoscopy) as well as the acceptance of this new technique within test group.

## Methods

### Study population

Both groups, test and control group (*N* = 48; males: 13, females: 35) were composed of 2nd year to 5th year medical students from the RWTH Aachen University, Aachen, Germany. They were randomized into equally sized (*N* = 24) test and control groups by using a random number generator (Randlist Version 1.2, DatInf GmbH, Tübingen, Germany). According to our inclusion and exclusion criteria, students should neither have a diagnosed impairment in binocular vision nor performed a bronchoscopy before. In addition, they should have basic knowledge in anatomy and internal medicine.

Since the effects by the use of ENB were not foreseeable, no power could be calculated. The sample size was limited by the number of volunteers available.

### Study protocol

**Fig. 1:**
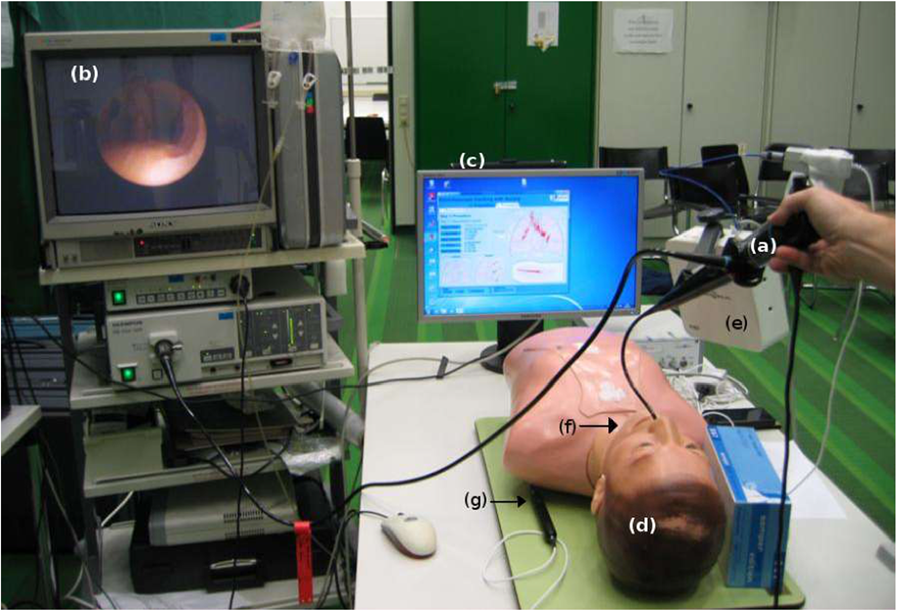
Experimental Setup. **a** Fiberoptic bronchoscope Olympus BF Type P40 (Olympus K.K., Tokyo, Japan) inserted in the phantom. **b** Video centre Olympus OTV-F3. **c** Graphical user interface of the self-developed software showing the path and position of the bronchoscope’s tip in the lung. **d** Training phantom Broncho Boy CLA 9 (Coburger Lehrmittelanstalt, Coburg, Germany). **e** Planar field generator, (**f**) reference coil (not visible) and (**g**) calibration pointer of the Aurora electromagnetic tracking system (Northern Digital Inc., Waterloo, Ontario, Canada)

The study protocol and design were approved by the local ethics committee of the University Hospital Aachen (EK 175/12). All subjects were asked to perform a systematic diagnostic bronchoscopy on the training phantom Broncho Boy CLA 9 (Coburger Lehrmittelanstalt, Coburg, Germany) using the fiberoptic bronchoscope Olympus BF Type P40 (Olympus K.K., Tokyo, Japan) with light source Olympus CLV U20 and video center Olympus OTV-F3. Note that in a systematic diagnostic bronchoscopy all five lobes must be closely examined. In addition, the Aurora electromagnetic tracking system (Northern Digital Inc., Waterloo, Ontario, Canada) was used to track the position of the bronchoscope’s tip. In short, this guidance system used an electromagnetic field to determine the location of the bronchoscope’s tip, which was embedded with sensor coils [27]. The planar field generator of Aurora was mounted slightly above the thorax of the training phantom and the sensor was positioned at the tip of the bronchoscope by pushing it through the working channel. The sensor was calibrated by defining specific points on the phantom’s thorax. Figure 1 illustrates the current experimental setup. The control group performed a conventional bronchoscopy using only the image provided by the camera of the bronchoscope for guidance. The path of the bronchoscope’s tip was recorded in parallel. The test group had additionally the information provided by the Aurora electromagnetic tracking system, which was displayed in monitor (Fig. 1c). During the experiments, the volunteers were observed by a trained study supervisor, who annotated in parallel their accomplishments and failures.

The aim of each simulated systematic bronchoscopy was first to locate and examine all five bronchial lobes while trying to minimize the procedure time. If the volunteers were not able to complete the task in 10 min, the experiment was interrupted. To equalize inter-individual differences, a brief (approximately 10 min) standardized introduction to lung anatomy and indications for a (systematic) bronchoscopy along with an instruction for handling the bronchoscope were given. Table 1 describes the content of the standardized introduction. Furthermore, an anatomic drawing of the bronchial tree up to the segmental ramifications was provided during the introduction in order to explain the lung anatomy.

**Table 1:** Content of the standardized introduction

Theoretical background	a. Lobes and segmental lung anatomy b. Definition of systemic bronchoscopy
Correct handling of the bronchoscope	a. Insertion of the bronchoscope via mouth vs. nostril b. Keep the bronchoscope’ fibres stretched c. Control via lever for flexing and extending the distal tip d. Wrist rotation around the bronchoscope’s axis e. Moving forward under vision only f. Central position of bronchoscope for optimal vision and to avoid (wall) trauma g. Maintain spatial orientation (ventral vs dorsal, left vs right)
Task	a. Performance of a systematic bronchoscopy b. Exploration and examination of all five bronchial lobes c. Duration: 10 min

The number of lobes examined was defined as the primary outcome parameter and the examination time as one of the secondary outcome parameters. In addition, before and after the systematic bronchoscopy all volunteers were asked to fill out a self-assessment questionnaire with 16 questions (Additional file 1). It aimed to assess background knowledge, experience as well as the feedback of the subjects about the study. The test group was additionally asked to rate the level of assistance provided by the bronchoscopy guidance system. The answers were given as a five-level Likert scale (positively skewed).

### Graphical user Interface

**Fig. 2:**
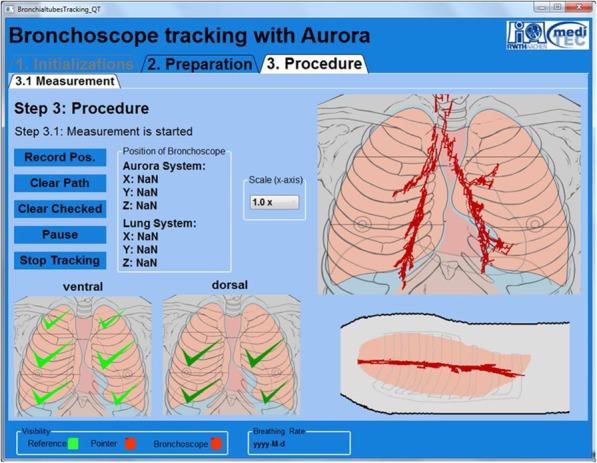
Graphical user interface of the self-developed software. The tracking path, displayed by the red dots, describe the recorded movement after a completed systemic bronchoscopy. Frontal and sagittal plane can be visualized

**Fig. 3:**
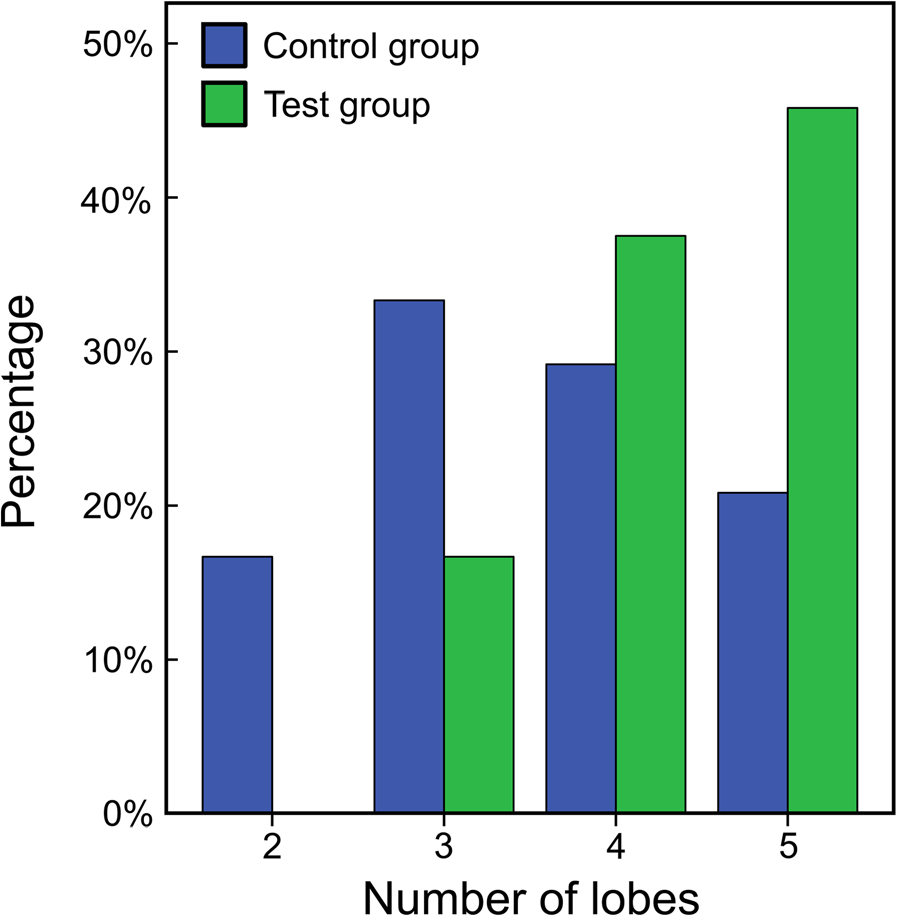
Number of lobes examined and the respective percentage of subjects who accomplished it. The plot compares both groups control and test group

A self-developed software using C# was used to record the path while tracking. Its graphical user interface (GUI) is displayed in Fig. 3. It permits to plot the current Cartesian coordinates (x,y,z) of the bronchoscope’s tip during the measurement. Moreover, the GUI is capable of displaying the whole analysis path, as represented by the red dots in Fig. 2 - right. For better spatial orientation and comprehension, the plot area was underlaid with a picture of a thorax (frontal and sagittal plane).

### Statistical analysis

For statistical analysis, the answers to the questionnaire, the procedure time as well as the accomplished tasks were considered. For each single examined lobe one point was given. The total number of examined lobes was also considered with a maximum score of 5 points (1 point for lobe).

Due to the very limited number of subjects, not normal distribution was considered. As a result, the following statistical methods were used:

- *Chi-square test*: for categorical data (e.g. if a specific lobe was found or not);
- *Mann-Whitney U test*: for numerical data (time, number of lobes examined, etc.).

To find correlation between parameters the Spearman rank correlation was used. A *p* value < 0.05 was defined statistically significant. For statistical analysis SPSS Statistics Version 23 (IBM Corporation, Armonk, New York, USA) was used.

## Results

**Fig. 4:**
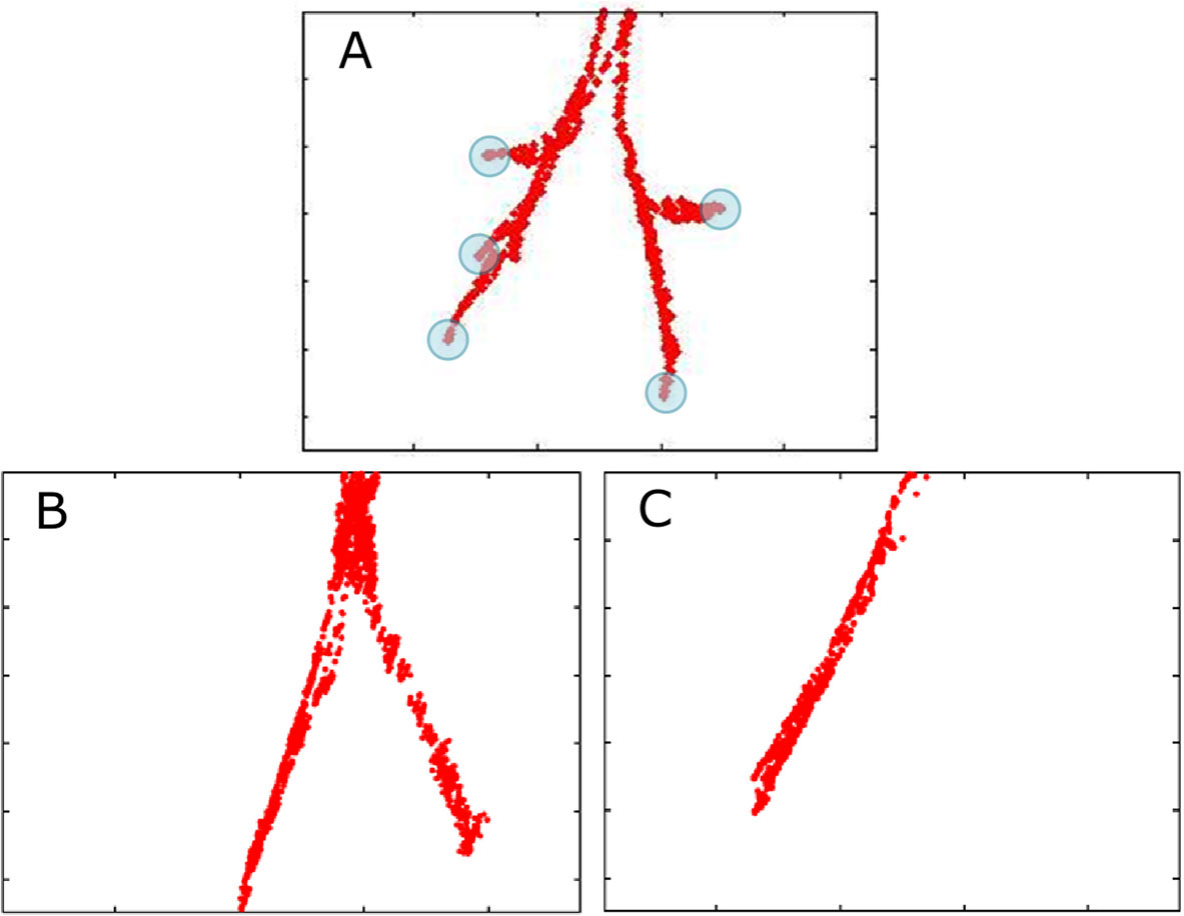
Three examples of the tracking results. **a** Correct bronchoscopy. All five lobes were examined (blue circles). (B and C) Incomplete bronchoscopies. In (**b**) only the left and right lower lobes were examined. In (**c**) the volunteer only assessed the right middle and lower lobe

**Fig. 5:**
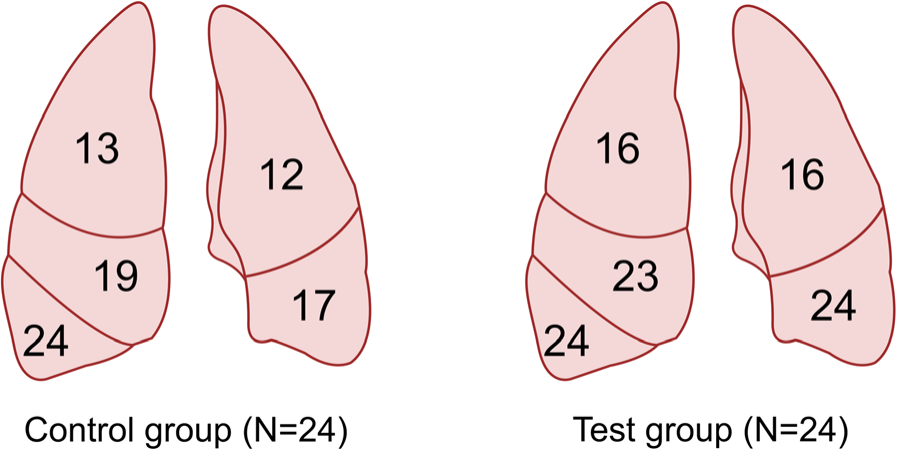
Illustration representing the number of volunteers that assessed a determined lung lobe for both groups, control and test group

As previously referred, all students were randomly divided into two sub-groups: control (*N* = 24; males: 8, females: 16) and test group (N = 24; males: 5, females: 19). The questionnaire has shown that all students performed a bronchoscopy for the first time in this study. The Chi-Quadrat-Test demonstrated a significant difference between control and test group regarding examination of the left lower lobe X (1)=8.195, *p* = 0.004. For the right middle lobe the difference was obvious but not statistically significant X (1)=3.048, *p* = 0.081. The difference between groups for the number of examined lobes was statistically significant as well (U = 168, *p* = 0.009). Figure 3 shows that in general the test group examined more lobes than the control group within the 10 min time slot. The Spearman’s rank correlation demonstrated that the number of lobes examined did not correlate with the time of procedure. In Fig. 4 the tracking results for one successful (A) and two unsuccessful (B, C) bronchoscopies are illustrated. Figure 5 shows for each lung lobe the number of students that assessed it. Regarding procedure time (control-group: 6.86 ± 2.09 min; test-group: 7.43 ± 2.04 min) and knowledge of lung anatomy no differences were found. However, the Mann-Whitney U Test showed a significant difference for the number of semesters studied. The control group was composed of students in higher semesters. After the experiment, the test group was more confident, having analyzed the entire lung (U = 160.5, *p* = 0.007).

Regarding the tracking system, the test group found the tracking helpful for orientation (91.3% of the volunteers agreed at least slightly) and agreed that it can improve bronchoscopy training in the future (95.8% of the volunteers agreed at least slightly). Furthermore, mostly volunteers considered that the bronchoscopy guidance system permitted to obtain a better overall impression of the lung anatomy (79.1%). In general, after this study, they feel more confident (91.7%) and they believe that during the next study/examination they will orient themselves faster (91.6%). Lastly, 95.8% of the volunteers would like to use this or other guidance system during the next bronchoscopy.

## Discussion

In general, the results of this study demonstrate that the use of a bronchoscopy guidance system improved the performance of the students during a systematic diagnostic bronchoscopy.

As demonstrated in Fig. 3, the test group examined more lobes than the control group within the 10-min time slot. For neither group, the number of lobes examined correlated with the time of procedure. Regarding the control group, this indicates that only the image of the bronchoscope does not offers enough information to the subjects, especially in the case of unexperienced students or novice bronchoscopists, who can easily oversee certain parts of the lung. In the test group, approximately 54% of the volunteers did not get to examine the five lobes. This was mainly due to (1) lack of time, (2) interpretation issues, and (3) software problems. Some students had difficulties interpreting the tracking path shown on the monitor. As shown in Fig. 2, the path does not match the picture of the thorax; this was often misleading, particularly when subjects were under stress while performing their first bronchoscopy. Each time a lobe was successfully examined, the tracking system detected it and set a check in the left lower part of the GUI (Fig. 2). Unfortunately, in some cases, an examination was considered valid shortly before the final target was achieved.

As mentioned previously, the Chi-Quadrat-Test demonstrated a significant difference between control and test groups regarding the examination of the left lower lobe (*p* = 0.004) as well as an obvious but not statistically significant difference for the right middle lobe (*p* = 0.081). Figure 5 complements the results. Accordingly, the bronchoscopy guidance system improved the performance with respect to these lobes. All 48 subjects found the right lower lobe without problems. However, both groups demonstrated difficulties in assessing the upper right and left lobes as shown in Fig. 5. This might be due to the lung anatomy: the upper lobe orifices are just below the carina and to reach these regions the tip of the bronchoscope must be turned upwards (headwards) with maximum flection (see Figs. 3 and 4(a)). This task is particularly difficult for trainees handling a bronchoscope for the first time. Figure 4(b and c) shows two examples where the subjects (in this case from the control group) were unable to manipulate the tip of the bronchoscope. An incorrect interpretation of the tracking path and software problems were further concomitant factors. It is a limitation that the outcome measure is a crude measure of competence in bronchoscopy. In clinical use it is not enough to be able to maneuver the scope to all five lobes, actually all 19 segments of the lung must be identified. But the identification of all lobes is the first step.

Regarding procedure time, no differences were found. The bronchoscopy guidance system does not decrease the examination time per se. It improves on the contrary the examination performance and ensures the trainee/bronchoscopist that an analysis of the whole lung was carried out. Indeed, after the experiment the test group was more confident, having analyzed the entire lung (U = 160.5, *p* = 0.007). At this point a more detailed and clinical orientated outcome measure, not only maneuvering the bronchoscope through lung lobes, is required for future research.

In general, the questionnaire demonstrated a positive feedback from the participants though it needs to be mentioned that these are rather subjective opinions and limit an objective evaluation of the outcome. The majority of the test group considered the tracking helpful for orientation and agreed that it can improve bronchoscopy training in the future. By using the bronchoscopy guidance system, the subjects felt more confident and believe that they are likely to orient themselves faster in the next study/examination. More importantly, 95.8% of the volunteers admitted that they would like to have a guidance system during the next bronchoscopy. However, 20.9% of the students think that the current system does not offer a good overall impression of the lung anatomy yet. A 3D reconstruction of the lung could be a possible solution to this problem [28, 29]

As mentioned before, such tool is not only important for orientation, but it also gives the most important feedback, i.e. if a complete and correct examination was performed, which is essential for an improved performance [30]. Although the volunteers think that some improvements are still necessary, they agreed unanimously that they would like to use a guidance system during the next bronchoscopy. This indicates that the system has not only a great potential for training but can also play an important role in clinical routine. Looking back, it would have been important to have qualitative data on the differences in learning experiences between the two groups. These could also be included in the questionnaire in future studies.

Colt et al. [31] and Moorthy et al. [32] have compared the medical field to the airline industry. In the latter, training is costly but instrumental in avoiding errors. In the medical field, manual and technical skills as well as experience are also crucial, especially in case of invasive procedures such as flexible fiberoptic bronchoscopy. Until recently, a conventional training was performed on real patients under supervision. However, of course this represents an increased risk for erroneous diagnoses, patient discomfort and procedure-related morbidities. The authors believe that novel technologies, such as guidance systems and virtual reality, improve significantly the accuracy and dexterity of novices. These are particularly interesting for unsupervised learning/practice. We fully share these authors views. Indeed, our work demonstrates a good acceptance of the tracking guidance system among students. However, we are aware that several improvements regarding software and hardware of the tracking guidance system are still necessary.

## Conclusions

The current work demonstrates that a bronchoscopy guidance system is an efficient proof-of-concept for training of novices. In our opinion, such a tool might improve the overall quality of lung examination. With a simulation environment, novices get the opportunity to individualize the training, to repeat and to correct errors. The guidance system provides the trainees with the additional improving their orientation as well as their overall impression of the lung anatomy. By offering a real-time feedback, the user always knows the actual position of the bronchoscope’s tip. According to our study, this extra information permitted improving the overall performance of the volunteers. Furthermore, they felt more confident after having accomplished their task. Within this context, the guidance system might be especially interesting for unsupervised learning. Finally, our work showed a distinct acceptance of the tracking system. Though in clinical routine physicians and novices should be able to identify the lung anatomy without a tracking system, this guided training provides a great potential in unsupervised learning and faster improving performances in clinical routine.

## Supplementary information

**Additional file 1.** Self-assessment questionnaire.

## Data Availability

The datasets used and/or analysed during the current study are available from the corresponding author on reasonable request. An SPPS-File containing the dataset is also available at Open Science Framework (http://www.osf.io) as project with Code “pn7kh” or follow this link: https://osf.io/pn7kh/.

## Abbreviations

ENB: Electromagnetic Navigation Bronchoscopy
GUI: graphical user interface
