# Supplementary material for: Evaluation of a bronchoscopy guidance system for bronchoscopy training, a randomized controlled trial
Source: BMC Med Educ. 2019 Nov 21;19:430. doi: 10.1186/s12909-019-1824-3 (PMC6868732; doi:10.1186/s12909-019-1824-3)
Supplement: Supplementary file 1 — Additional file 1. Self-assessment questionnaire. [file 12909_2019_1824_MOESM1_ESM.pdf]

Questionnaire - Bronchoscopy guidance system study

1st Part

1. Age: _______ years

2. Gender: ☐ Male ☐ Female

3. Level of medical training
   - ☐ Medical Student (____ semester)
   - ☐ Resident physician - Anesthesiology (____ year)
   - ☐ Consultant physician - Anesthesiology
   - ☐ Senior physician – Anesthesiology

4. How many bronchoscopies have you already performed?
   - ☐ 0
   - ☐ 1-5
   - ☐ 6-20
   - ☐ More than 20

5. How is your lung anatomy knowledge?
   - ☐ Very Poor
   - ☐ Poor
   - ☐ Acceptable
   - ☐ Good
   - ☐ Very Good

6. How is your experience regarding bronchoscopy?
   - ☐ Very Poor
   - ☐ Poor
   - ☐ Acceptable
   - ☐ Good
   - ☐ Very Good

7. Are you confident having examined the entire lung in previous bronchoscopies?

- ☐ Not confident at all
- ☐ Unconfident
- ☐ Somewhat confident
- ☐ Confident
- ☐ Absolutely confident

Questionnaire - Bronchoscopy guidance system study

2nd Part (Feedback after performing the bronchoscopy)

8. Are you confident having examined the entire lung?
   - ☐ Not confident at all
   - ☐ Unconfident
   - ☐ Somewhat confident
   - ☐ Confident
   - ☐ Absolutely confident

9. How big is the probability of you having caused a mucosal wall trauma?
   - ☐ Very low
   - ☐ Below Average
   - ☐ Average
   - ☐ Above Average
   - ☐ Very High

10. Did you feel stressed during the bronchoscopy?
    - ☐ Never
    - ☐ Seldom
    - ☐ Sometimes
    - ☐ Often
    - ☐ Almost always

Only applicable for Group A test group and Group B

11. Did the bronchoscopy guidance system improved your orientation?
    - ☐ Disagree Strongly
    - ☐ Disagree
    - ☐ Agree Slightly
    - ☐ Agree
    - ☐ Agree Strongly

12. Do you think that such system could improve bronchoscopy training in the future?
    - ☐ Disagree Strongly
    - ☐ Disagree
    - ☐ Agree Slightly
    - ☐ Agree
    - ☐ Agree Strongly

13. By using the bronchoscopy guidance system, did you get a better overall impression of the lung anatomy?
    - ☐ Disagree Strongly
    - ☐ Disagree
    - ☐ Agree Slightly
    - ☐ Agree
    - ☐ Agree Strongly

14. Do you expect to feel more confident during your next bronchoscopy (independently of the availability of a tracking system)?
    - ☐ Disagree Strongly
    - ☐ Disagree
    - ☐ Agree Slightly
    - ☐ Agree
    - ☐ Agree Strongly

15. Do you expect to orient yourself faster during the next bronchoscopy (independently of the availability of a tracking system)?

    - ☐ Disagree Strongly
    - ☐ Disagree
    - ☐ Agree Slightly
    - ☐ Agree
    - ☐ Agree Strongly

16. Would you like to use this or other guidance system during your next bronchoscopy?

    - ☐ Yes
    - ☐ No
